# WASH to control COVID-19: A rapid review

**DOI:** 10.3389/fpubh.2022.976423

**Published:** 2022-08-11

**Authors:** Mahalaqua Nazli Khatib, Anju Sinha, Gaurav Mishra, Syed Ziauddin Quazi, Shilpa Gaidhane, Deepak Saxena, Abhay M. Gaidhane, Pankaj Bhardwaj, Shailendra Sawleshwarkar, Quazi Syed Zahiruddin

**Affiliations:** ^1^Division of Evidence Synthesis, School of Epidemiology and Public Health, Jawaharlal Nehru Medical College, Datta Meghe Institute of Medical Sciences, Wardha, India; ^2^Division of Reproductive, Maternal and Child Health, Indian Council of Medical Research Headquarters, New Delhi, India; ^3^Department of Radiology, Jawaharlal Nehru Medical College, Datta Meghe Institute of Medical Sciences, Wardha, India; ^4^School of Epidemiology and Public Health, Jawaharlal Nehru Medical College, Datta Meghe Institute of Medical Sciences, Wardha, India; ^5^Department of Medicine, Jawaharlal Nehru Medical College, Datta Meghe Institute of Medical Sciences, Wardha, India; ^6^Department of Public Health, Indian Institute of Public Health Gandhinagar, Gandhinagar, India; ^7^Jawaharlal Nehru Medical College, Datta Meghe Institute of Medical Sciences, Wardha, India; ^8^Department of Community Medicine, All India Institute of Medical Sciences, Jodhpur, India; ^9^Faculty of Medicine and Health, Sydney Medical School, The University of Sydney, Camperdown, NSW, Australia; ^10^Centre for Global Evidence Synthesis Initiative (GESI), School of Epidemiology and Public Health, Jawaharlal Nehru Medical College, Datta Meghe Institute of Medical Sciences, Wardha, India

**Keywords:** COVID-19, water sanitation and hygiene (WASH), interventions, SARS, public health, sanitation, hygiene

## Abstract

**Background:**

Preventive public health has been suggested as methods for reducing the transmission of COVID-19. Safety and efficacy of one such public health measure: WASH intervention for COVID-19 has not been systematically reviewed. We undertook a rapid review to assess the effect of WASH intervention in reducing the incidence of COVID-19.

**Methods:**

We conducted searches in PubMed, MEDLINE, and EMBASE. We undertook screening of studies in two stages and extracted data and assessed the quality of evidence for the primary outcome using GRADE recommendations.

**Main results:**

We included a total of 13 studies with three studies on COVID-19 and 10 on SARS. The study found that hand washing, sterilization of hands, gargling, cleaning/shower after attending patients of COVID-19, or SARS was protective. Evidence also found that frequent washes can prevent SARS transmission among HCWs. However; one study reported that due to enhanced infection-prevention measures, front-line HCWs are more prone to hand-skin damage. The certainty of the evidence for our primary outcome as per GRADE was very low. We did not find any studies that assessed the effect of WASH on hospitalizations, and mortality due to COVID-19. Also; we did not find any study that compared WASH interventions with any other public health measures.

**Conclusions:**

Current evidence of WASH interventions for COVID-19 is limited as it is largely based on indirect evidence from SARS. Findings from the included studies consistently show that WASH is important in reducing the number of cases during a pandemic. Timely implementation of WASH along with other public health interventions can be vital to ensure the desired success. Further good-quality studies providing direct evidence of the efficacy of WASH on COVID-19 are needed.

## Introduction

In the last month of 2019, a novel coronavirus, called SARS-CoV-2 emerged in China and caused an outbreak of coronavirus disease 2019 (COVID-19) (1). By January 30, 2020, the World Health Organization (WHO) declared COVID-19 as a Public Health Emergency of International Concern (2, 3). The rise in the number of cases was contributed by person-to-person transmission in family homes, hospitals, and community and intercity (4–6). As of now, there is no known specific, effective, proven, pharmacological treatment. Slowing down the spread of COVID-19 through public health and social measures currently seem the mainstay of tackling the pandemic (7, 8). However, it would be very difficult to maintain the lockdown of institutions and public places and restrict trade and travel indefinitely.

Preventive public health measures such as isolation of cases, quarantine, hand hygiene practices, masks, physical distancing (including lockdown), quarantine, personal protective equipment (PPEs), and other workplace interventions have been suggested as methods for reducing the transmission of COVID-19 (8–10). WASH is the collective terminology for Water, Sanitation, and Hygiene interventions. As these three words are interdependent, these are bracketed together (5). The facility of safe water, sanitation, and hygiene are vital in safeguarding health epidemics of communicable diseases, including the current COVID-19 pandemic (5).

Evidence in hand shows that SARS-CoV-2 is transmitted *via* respiratory droplets (11). Droplets usually land on surfaces where the virus can remain viable. Thus, the area around an infected COVID-19 patient can act as a source of contact transmission. Once hands come in direct contact with the contaminated surface, the contaminated hands can cause self-inoculation by touching the mucous membranes of the nose, mouth, or eyes (5). The contaminated hands can also transmit the virus to another surface, which further facilitates indirect transmission. WASH intervention including hand hygiene is very important in reducing the chances of this self-contamination (12), subsequent nasal inoculation thereby curtailing the spread of the COVID-19 (13). Though SARS-CoV-2 has not been detected in drinking water, conservative methods of water treatment such as filtration and disinfection can deactivate the SARS-CoV-2 as other types of coronaviruses were found to be inactivated by chlorination and disinfection with ultraviolet light (5, 14).

The safety and efficacy of WASH intervention for COVID-19 have not been systematically reviewed. Therefore, we undertook this rapid review to assess the efficacy of WASH interventions in reducing the incidences of COVID-19. The review also sought to assess the effectiveness of WASH intervention in reducing mortality due to COVID-19 and explore any variations in the effectiveness of WASH in different settings.

## Rationale

Evidence in hand shows that SARS-CoV-2 is transmitted *via* respiratory droplets. The contaminated hands can also transmit the virus to another surface, which further facilitates indirect transmission. The area around an infected COVID-19 patient can act as a source of contact transmission. Effectiveness of WASH intervention in reducing mortality due to COVID-19 and explore any variations in the effectiveness of WASH in different settings. SARS-CoV-2 has not been detected in drinking water, conservative methods of water treatment such as filtration and disinfection can deactivate the SARS-CoV-2 as other types of coronaviruses were found to be inactivated by chlorination and disinfection with ultraviolet light. Findings from the included studies consistently show that WASH is important in reducing the number of cases during a pandemic. Timely implementation of WASH along with other public health interventions can be vital to ensure the desired success.

## Methods

This rapid review has been prospectively registered in Prospero (Registration Number: CRD42020179663) (15). Though we adhered to PRISMA (16) guidelines throughout this manuscript; we curtailed the systematic review methods and adopted the following shortcuts recommended in methodology to undertake this rapid review:

- We restricted the number of comparisons and outcomes.
- We did not undertake searches of gray literature; or contact experts for on-going studies or any authors for missing data.
- During the screening of studies for eligibility criteria, the second reviewer checked 30% of the excluded records in the first phase and 100% of records in the second phase of screening.

### Criteria for considering studies for this review

Pre-specified eligibility criteria were as follows:

#### Study design

We included a broad range of study designs such as cohort studies, case-control studies, time series, interrupted time series, and mathematical modeling studies. We excluded case reports, case series and case studies in this rapid review.

#### Population

We included studies reporting the efficacy of WASH interventions (irrespective of study setting) in contacts of suspected or confirmed cases, individuals residing in areas with the rising trend in cases, or individuals traveling from areas where COVID-19 outbreaks were declared. We considered “Outbreaks” as an “occurrence of disease cases in excess of normal expectancy” (17). We included studies irrespective of age, gender, race/ethnicity of individuals, or presence of chronic/comorbid conditions. As a piece of indirect evidence for COVID-19, we also included studies on a similar condition SARS. We excluded studies on individuals with symptoms suggestive of COVID-19 such as Middle-East Respiratory Syndrome (MARS) infections, and studies on asymptomatic individuals with a history of exposure to other organisms causing other respiratory infections.

#### Intervention

We included studies that assessed the efficacy of different types of WASH (Water, Sanitation, and Hygiene) interventions either as a single measure or in combination with other public health measures like quarantine, personal protective equipment's (PPEs), physical distancing including lockdown, other workplace interventions, training; etc. We defined WASH intervention as per the earlier Cochrane reviews on WASH (18). We included different components and types of WASH interventions irrespective of setting (community or hospital). We excluded studies that have reported the efficacy of WASH interventions in combination with other public health measures related to travel.

#### Comparator(s)

We include studies that compare:

1. WASH interventions with no WASH interventions.
2. WASH interventions vs. any other public health measures (without WASH interventions) like quarantine of individuals or a community, PPEs, physical distancing including lockdown, other workplace interventions; etc.

#### Outcome(s)

We reported the following outcomes:

Primary outcome:

1. Number of COVID-19 cases (reported as per clinical or lab diagnosis by the authors of included studies).

Secondary outcomes:

1. Hospitalizations (reported as individuals hospitalized for symptoms suggestive of COVID-19 by the authors of included studies).
2. Mortality due to COVID-19 (reported as deaths due to COVID-19 by the authors of included studies).
3. Adverse events (reported as adverse events by the authors of included studies).

We reported data on time points as reported in studies.

#### Search methods for identification of studies

An information specialist designed and conducted literature searches systematically, which were verified by a content expert (SZQ) and peer-reviewed independently. The information specialist undertook searches in MEDLINE, CENTRAL, and EMBASE. We also searched for the WHO Global Index Medicus (https://www.who.int/library/about/The_Global_Index_Medicus/en/). The detailed search strategies are presented in Supplementary Table 1. Additionally; we screened the reference lists of the included studies and related systematic reviews for identifying potentially relevant studies. As we are not expecting to find any conference abstracts as the conferences have been postponed/rescheduled because of the COVID-19 pandemic, we did not search for conference abstracts.

#### Screening and selection of studies

We exported all the records identified through a systematic literature search to the Rayyan web-app (19) and removed the duplicates. We undertook screening of records in two stages. In the first stage, one reviewer with expertise in systematic reviewing (MNK) screened all titles and abstracts for eligibility as per the pre-defined inclusion and exclusion criteria and a second reviewer (DS) checked 30% of the excluded records. One reviewer (MNK) then reviewed full texts of all the records deemed eligible in the first stage of screening and the second reviewer (DS) checked all the excluded records. We resolved disagreements by consensus or by involving a third senior reviewer (AS). We recorded all decisions taken during screening and outlined the list of excluded studies separately. We excluded studies published in languages other than English or Chinese. We included Chinese studies only if abstracts or summaries are available in English.

#### Data extraction

One reviewer (SZQ) conducted data extraction with a pilot-tested form using Excel and a second reviewer (AG) verified the same. We recorded the following data:

- Study design
- Setting
- Participant characteristics
- Intervention characteristics
- Comparator characteristics
- Outcomes assessed
- Numerical data for outcomes of interest
- For modeling studies, we additionally extracted data for the type of model, and data source.

#### Risk of bias assessment

One reviewer (MNK) conducted a Risk of Bias (RoB) assessment and a second reviewer (PB) verified the same. The RoB was assessed with “Tool to assess the risk of bias in case-control studies” (20) and “Tool to assess the risk of bias in cohort studies” (21). We resolved discrepancies by discussion and involving a third reviewer (AS). Due to time constraints, we did not contact the authors to seek missing information.

#### Data synthesis

We had planned to synthesize data by conducting meta-analyze only if participants, interventions, comparisons and outcomes are judged to be sufficiently similar and relevant. However; we found diverse types of participants, interventions, methods of measurement, manner of reporting of outcomes in included studies, and subsequent heterogeneity. Hence; we did not pool the results of the included studies in meta-analysis and rather preferred to present a qualitative description of these studies with supporting tables as narrative synthesis. We had planned to quantify heterogeneity by using *I*^2^ statistics and explore possible causes of heterogeneity among study results by undertaking subgroup analysis for the primary outcome in terms of different age and presence/ absence of chronic or comorbid conditions. We had planned to assess reporting biases by inspecting funnel plots for asymmetry (If more than ten studies included in meta-analysis). However; due to lack of studies, we were not able to do so. To explore the possible influence of covariates, we had planned to undertake subgroup analyses for primary outcome stratified by age, and presence/absence of chronic/comorbid conditions. As we did not undertake any meta-analyses, subgroup analysis was not possible. We had planned to conduct sensitivity analyses by excluding studies rated as “high risk” of bias. As we did not undertake any meta-analyses, sensitivity analyses was not possible.

#### Assessment of the certainty of the evidence

One reviewer (AG) assessed the certainty of the evidence for the primary outcome using GRADE (Grading Quality of Evidence and Strength of Recommendations) (22) recommendations and presented the results in a summary of findings table (Table 1). GRADE uses four categories to classify the certainty of evidence. A “high” certainty rating of a body of evidence means that we were very confident that the estimated effect lies close to the true effect; “moderate” certainty means we assume the estimated effect is probably close to the true effect; a “low” certainty rating suggests that the estimated effect might substantially differ from the true effect; and “very low” certainty means that the estimated effect is probably markedly different from the true effect. Observational studies start with moderate quality of evidence and are downgraded as per assessments of RoB, indirectness, inconsistency, imprecision, and publication bias.

**Table 1:** Summary of findings for the primary outcome (Number of cases of COVID-19).

**WASH intervention in combination with other public health measures compared to no intervention for reducing the number of cases of COVID-19**					
Patient or population: Reducing the number of cases of COVID-19					
Setting: Hospital					
Intervention: WASH intervention in combination with other public health measures					
Comparison: No intervention					
**Outcomes**	**No of participants (studies) follow up**	**Certainty of the evidence (GRADE)**	Relative effect*(95% CI)	**Anticipated absolute effects**	
				**Risk with no intervention**	**Risk difference with WASH intervention in combination with other public health measures**
Number of cases of COVID-19 assessed with: Any criteria for labeling a confirmed case of COVID-19 as reported by the authors	0 cases 72 controls (1 observational study)	⊕○○○ Very low^a, b^	Not estimable	Low	
				0 per 1,000	0 fewer per 1,000 (0 fewer to 0 fewer)

* The risk in the intervention group (and its 95% confidence interval) is based on the assumed risk in the comparison group and the relative effect of the intervention (and its 95% CI).

CI, Confidence interval.

GRADE Working Group grades of evidence.

High certainty: We are very confident that the true effect lies close to that of the estimate of the effect.

Moderate certainty: We are moderately confident in the effect estimate: The true effect is likely to be close to the estimate of the effect, but there is a possibility that it is substantially different.

Low certainty: Our confidence in the effect estimate is limited: The true effect may be substantially different from the estimate of the effect.

Very low certainty: We have very little confidence in the effect estimate: The true effect is likely to be substantially different from the estimate of effect.

^a^Downgraded once for study design.

^b^Downgraded twice for imprecision due to sparse data and low participant numbers.

## Current state of knowledge

### Results of the search

**Figure 1:**
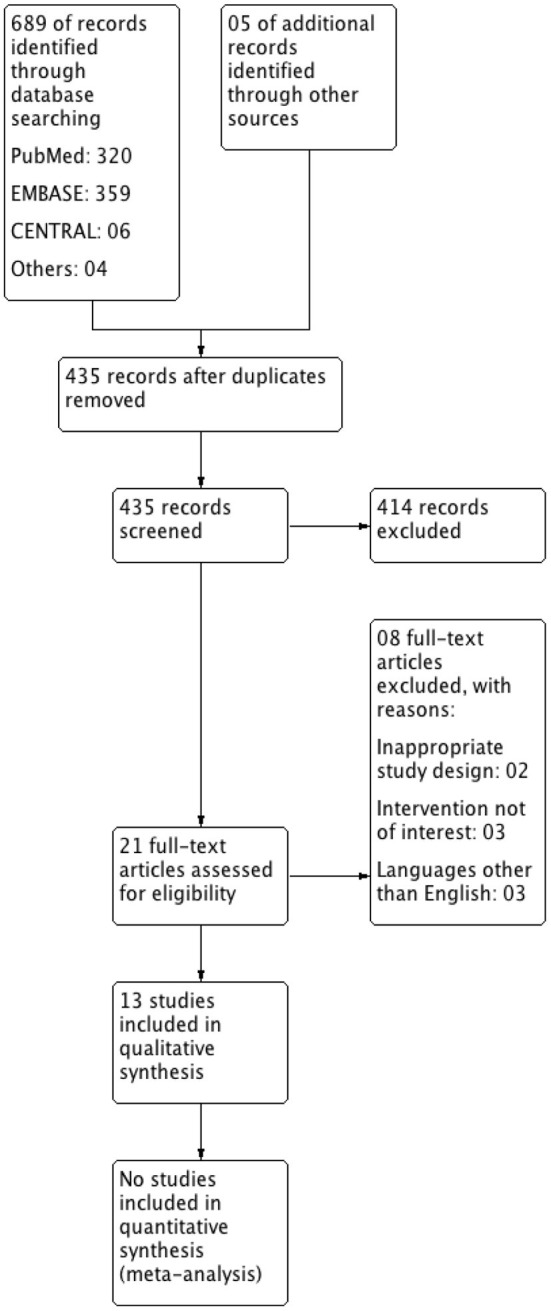
PRISMA flow diagram for inclusion of studies.

The PRISMA flow diagram (Figure 1) provides an overview of the study selection process. We identified 689 records from electronic searches and five records from other sources. All 435 records that remained after removal of duplicates were screened initially based on title and abstract during which we excluded 414 records and 21 potentially relevant records were subsequently screened based on full-text. Thirteen records met the eligibility criteria and were included in this review. However; due to diverse types of interventions, methods of measurement, and manner of reporting of outcomes and subsequent heterogeneity; we did not undertake quantitative synthesis. We have recorded the reasons for the exclusion of seemingly related studies in a separate table of excluded studies (Supplementary Table 2).

### Included studies

We have presented the characteristics of the studies that met the inclusion criteria in “Characteristics of included studies table” (Table 2) and the results of each study in “Results of included studies table” (Tables 3–5). Our searches identified 13 relevant studies (Figure 1). Of these, three focused on COVID-19 (23–25) and 10 focused on SARS (26–33). All three studies addressing COVID-19 were case-control studies conducted in China (23–25). From the 10 studies focusing on SARS, nine were hospital-based case-control studies from China (26–32), Hong Kong (13, 27, 33), Taiwan (31), and Singapore (34), and one modeling study from Taiwan (35). All the participants in the included studies were HCWs. We did not find any study done in community settings on the general population. All the included studies focus on hygiene either alone or in combination with any other public health measures like quarantine of individuals or a community, PPEs, physical distancing, training, prophylactic medicines, other infection control, or workplace interventions; etc. We did not find any study done to assess the effectiveness of sanitation in controlling the pandemic. We did not find any studies that compared WASH interventions with any other public health measures (without WASH interventions) like quarantine of individuals or a community, PPEs, physical distancing including lockdown, other workplace interventions; etc. We found only studies that reported the adverse events related to WASH and no studies that assessed the effect of WASH on mortality, and hospitalizations.

**Table 2:** Characteristics of included studies.

**Author and country**	**Study design**	**Intervention**	**Outcome**
Ran et al. (25) China	Retrospective tertiary hospital-based setting	Qualified v/s unqualified handwashing Optimal v/s suboptimal hand hygiene Co-intervention: and/or PPE	Incident cases of SARS-CoV-2
Chen et al. (27) China	Retrospective hospital based study	Washing hands, nasal cavity and oral cavity (Frequency) Other factors assessed: PPE, training for SARS, ventilation, etc.	Cases of SARS
Lan et al. (24) China	Retrospective study Hospital-based	Handwashing with water and soap Other intervention: Other enhanced infection prevention measures	Adverse events of excessive handwash
Lau et al. (28) Hong Kong	Case-control study	Hand wash (Washed hands 11 or more times per day; Reference = 1–10 times/day) Other measures assessed: face masks, disinfections	Cases of SARS
Liu et al. (29) China	Case control study	Nose wash; Other protective measures: PPE, trainings, prophylactic medicine	Cases of SARS
Pei et al. (30) China	Case control study	Sterilization of hands, untouched hand-washing equipment's, gargling after contact with patients, cleaning oneself thoroughly when off duty were protective	Cases of SARS
Seto et al. (13) Hong Kong	Case-control hospital-based study (*n* = 5)	Hand-washing other factors assessed: masks, gloves and gown	Cases of SARS
Teleman et al. (30) Singapore	Case-control hospital-based study	Handwashing other protective measures assessed: N95 masks, gloves and gowns	Cases of SARS
Yen et al. (34) Taiwan	Modeling study by Structural Equation Modeling	Handwashing other protective measures assessed: other infection control measures	Cases of SARS
Yen et al. (33) Taiwan	Retrospective Hospital-based study	Traffic control bundle (Triage and diversion of patient before entrance to hospital, delineation of zones of risk, and hand disinfection at checkpoints between zones of risk)	Cases of SARS
Yin et al. (34) China	Case-control hospital-based study (*n* = 10)	Hand-washing and disinfection, gargle, shower, and changing clothing after work. Other protective measures assessed: PPE, avoidance from eating and drinking in ward, oseltamivir	Cases of SARS
Yu et al. (35) China and Hong Kong	Case-control hospital-based study (86 wards in 21 hospitals in Guangzhou and 38 wards in five hospitals in Hong Kong)	Washing facility Co-intervention: availability of changing facilities, minimum distance between beds of ≤ 1 m, use of an exhaust fan, use of high-flow-rate O_2_ mask, performance of resuscitation, staff working while experiencing symptoms, and a workload of <2 patients per one HCW	Incident cases of SARS

**Table 3:** Results of included studies table (Direct evidences on COVID-19 for primary outcome of Incident cases).

**SN**	**Study ID country**	**Study design and setting**	**Outcomes (as reported in studies)**	**Outcomes (interpretation)**	**Conclusion**
1.	Ran et al. (25) China	Retrospective study Hospital-based setting (Tertiary hospital single-center)	Qualified hand-washing (*n* = 22): infected: *n* = 04 Unqualified hand-washing (*n* = 50): infected: *n* = 24, RR (95% CI) = 2.64 (1.04–6.71), *P* ≤ 0.05 Qualified hand-washing v/s unqualified hand-washing: RR (95% CI): 0.38 (0.15–0.96), *P* = 0.04 Optimal hand hygiene before contact patients (*n* = 33): Infected: *n* = 06 Optimal hand hygiene after contact patients (*n* = 44): Infected: *n* = 11 Suboptimal hand hygiene before contact patients (*n* = 39): Infected: *n* = 22, RR (95% CI): 3.10 (1.43–6.73), *P* < 0.01 Suboptimal hand hygiene after contact patients (*n* = 28): Infected: *n* = 17, RR (95% CI): 2.43 (1.34–4.39), *P* < 0.01 Optimal handwash v/s Suboptimal handwash (after contact with patients): RR (95% CI) = 0.41 (0.23–0.74), *P* = 0.003	Optimal handwashing practices in HCWs reduces the risk of developing COVID-19 by 59% (*P* = 0.003) as compared to those with suboptimal handwash	HCWs working with suboptimal hand hygiene after contacting patients had a higher risk of COVID-19.
2.	Xu et al. (26) China	Prospective hospital-based study	Hand washing: 84 out of 206 (40.78%) complied to hand washing Disinfection: disinfection rate of environmental and medical supplies was 100% Cases of COVID-19: Nil	Though the compliance rate for hand hygiene was only 40.78%, no cases of COVID-19 were found	Refined management strategies for the prevention and control of nosocomial infections in HCWs.

**Table 4:** Results of included studies table (Indirect evidences on COVID-19 from SARS cases for primary outcome of Incident cases).

**SN**	**Study ID and country**	**Study design and setting**	**Outcomes (interpretation)**
1.	Chen et al. (27) China	Retrospective hospital-based study	Out of 748 frontline HCWs involved in care of SARS patients in two hospitals in China; 91 HCWs developed SARS.
2.	Lau et al. (28) Hong Kong	Case-control study	Out of 330 individuals, who developed SARS, 61 individuals washed hands 11 or more times per day. Out of 660 individuals, who did not develop SARS, 61 individuals washed hands 11 or more times per day.
3.	Liu et al. (29) China	Case control study	Out of 477 HCWs with SARS, 193 (40.46%) washed nose.
4.	Pei et al. (30) China	Case control study	Out of 147 infected SARS HCWs, 11 (7.5%) HCWs sterilized hands by iodine, 9 (6.9%) did gargling, and 109 (82%) cleaned themselves thoroughly after contact with patients. Out of 296 patients those did not develop SARS, 105 (39%) individuals sterilized hands by iodine, 38 (13.5%) individuals did gargling, and 261 (92.2%) cleaned themselves thoroughly after contact with patients.
5.	Seto et al. (13) Hong Kong	Case-control hospital-based study (*n* = 5)	Out of 13 infected SARS HCWs; 10 (77%) HCWs did handwashing and out of 241 patients that did not develop SARS, 227 (94%) HCWs did handwashing
6.	Teleman et al. (32) Singapore	Case-control hospital-based study	Out of 36 HCWs that developed SARS; 27 (75%) reported handwashing. Out of 50 HCWs that did not develop SARS, 46 (92%) HCWs reported handwashing.
7.	Yen et al. (36) Taiwan	Modeling hospitals (*n* = 48)	Installation of hand washing stations in ED was significantly associated with protection of HCWs from developing SARS
8.	Yen et al. (33) Taiwan	Retrospective Hospital-based study from epicenter of the SARS epidemic	Out of 19 hospitals with one or more HCWs with a nosocomial SARS infection, 6 (31.6%) hospitals installed hand-washing station in EDs, 10 (52.6%) hospitals had disinfectant solution available at hospital entrance, 5 (26.3%) hospitals had a set-up of hand-washing facilities around whole hospital, and 5 (26.3%) hospitals had a set-up alcohol dispensers at checkpoints between zones of risks. Out of 31 hospitals with no nosocomial SARS infection among HCWs, 28 (90.3%) hospitals installed hand-washing station in EDs, 30 (96.8%) hospitals installed hand-washing station in EDs, 20 (64.5%) hospitals had a set-up of hand-washing facilities around whole hospital, and 30 (96.8%) hospitals had a set-up alcohol dispensers at checkpoints for glove-on hand rubbing between zones of risks.

**Table 5:** Results of included studies table (Direct evidences on COVID-19 for secondary outcome of adverse events).

**SN**	**Study ID country**	**Study design and setting**	**Outcomes (as reported in studies)**	**Conclusion**
1.	Ran et al. (23–25) China	Retrospective study Hospital-based	321 out of 526 HCWs washed their hands >10 times per day reported more hand-skin damage [OR (95%CI) = 2.17 (1.38–3.43), *P* = 0.01] 113 out of 526 HCWs washed their hands ≤ 10 times per day Adverse events of excessive handwash (>10 times per day): itching 276 (52.5%) Out of 542 front-line HCWs for COVID-19; 526 (97%) reported hand-skin damage by enhanced infection-prevention measures.	Prevalence of skin damage of first-line HCWs managing COVID-19 is high. Longer exposure time was a significant risk factor for

HCWs, health care workers; OR, odds ratio; CI, confidence interval; MLR, multivariate logistic regression; ED, emergency departments.

### Effects of interventions

#### Comparison 1: WASH interventions vs. no WASH interventions

##### Effectiveness of WASH on the number of COVID-19 cases

**Figure 2:**
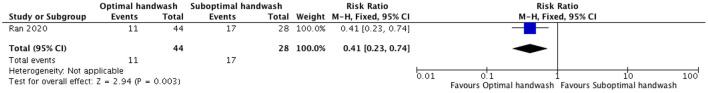
Forest plot showing effect of optimal handwash as compared to suboptimal handwash on the number of COVID-19 cases.

**Figure 3:**
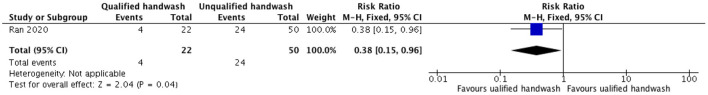
Forest plot showing effect of qualified handwash as compared to unqualified handwash on the number of COVID-19 cases.

We identified one retrospective (24) and one prospective study (25) from China that assessed the efficacy of WASH (handwash) and installation of rapid hand sanitizer stations, respectively, in reducing the cases of COVID-19 in HCWs attending to patients of COVID-19. We did not find any modeling study. We report the evidence narratively. The retrospective study from China (24) did not compare WASH intervention with no WASH intervention but compared optimal handwashing practices with sub-optimal handwashing practices, and qualified handwashing with unqualified handwashing. The study highlights the importance of optimal hand hygiene after coming in contact with COVID-19 patients by demonstrating that optimal handwashing practices in HCWs reduce the risk of developing COVID-19 by 59% (*P* = 0.003) as compared to those with suboptimal handwashing practices (Figure 2) (24). The study also compared qualified handwash with unqualified handwash and demonstrated that qualified handwash reduced the risk of developing COVID-19 by 62% (*P* = 0.04) as compared to unqualified handwash (Figure 3). Another prospective hospital-based study (25) from China was conducted on the efficacy of refined prevention and control management strategies including installation of rapid hand sanitizer stations in reducing the risk of COVID-19 cases among HCWs and individuals attending non-isolated areas in general hospitals such as outpatients, emergencies, wards, administrative offices with a high-risk of suspected cases. The study found that though the compliance rate for hand hygiene was only 40.78%, no cases of COVID-19 were found.

Overall, we included 10 studies including nine retrospective studies (13, 26–33), and one modeling study (34) that provided indirect evidence for the effect of WASH interventions in reducing the cases of SARS. The retrospective case-control studies used data from China (29–35), Hong Kong (13, 27, 33), Taiwan (32, 34), and Singapore (30) during or after the SARS outbreak in 2003. The modeling study (36) relied on data from SARS outbreaks in Taiwan.

##### Evidence on handwash

Six case-control studies and one modeling study assessed handwashing practices (13, 27, 30) and the availability of handwashing facility in hospitals (32–34) as a protective factor in controlling SARS infection in HCWs attending patients during the epidemic.

A study by Lau et al. (27) found that out of 330 individuals, who developed SARS, 61 individuals, washed hands 11 or more times per day, and that out of 660 individuals, who did not develop SARS, 61 individuals washed hands 11 or more times per day. The study concluded that washing one's hands more than 10 times a day is a significant protective factor and along with other public health, measures may have contributed substantially to the control of SARS epidemic in Hong Kong [Matched univariate OR (95% CI): 0.44 (0.31–0.63), *P* < 0.005]. Another study case-control hospital-based study conducted in five Hong Kong hospitals in 254 participants with exposure to 11 index patients of SARS during patient care (13). Out of 13 HCWs infected with SARS, 10 (77%), HCWs did handwashing and out of 241 patients that did not develop SARS, 227 (94%) HCWs did handwashing [OR (95%CI) = 5 (1–19), *P* = 0.022]. The study found that no staff became infected when they used hand-washing with PPE (13). A similar case-control hospital-based study by Teleman et al. (31) in Singapore undertaken to study the risk and protective factors for nosocomial transmission of SARS in a hospital during SARS outbreak found that out of 36 HCWs that developed SARS, 27 (75%) reported handwashing, while out of 50 HCWs that did not develop SARS, 46 (92%) reported handwashing [OR (95% CI) = 0.06 (0.007–0.5), *P* = 0.03]. The study found that hand washing after attending patients was found to be strongly protective with a 15-fold amelioration of odds (31).

Few studies (33, 35, 36) also assessed the protective effect of the installation of a handwashing facility in controlling SARS infection in HCWs. Yen et al. (36) conducted a Modeling study in 48 hospitals of Taiwan that provided hospitalization for 664 SARS patients. The study was conducted to determine the effectiveness of infection control measures (ICMs) by logistic regression and structural equation modeling (SEM); a quantitative methodology that can test a hypothetical model and validates causal relationships among infective control measures. Sixteen hospitals had episodes of infection of SARS in HCWs. The logistic regression analysis showed that the installation of handwashing stations in emergency departments was significantly associated with the protection of HCWs from developing SARS [OR (95%CI) = 1.07 (1.02–1.14), *P* = 0.012] (34). The study concluded that hospitals with better infection control measures are less likely to have HCWs acquiring SARS (34). Yen et al. (31) conducted a hospital-based retrospective study in one of the epicenters Taiwan to determine most effective factors in preventing nosocomial infections of HCWs during the 2003 SARS epidemic. Out of 19 hospitals with one or more HCWs with a nosocomial SARS infection, 6 (31.6%) hospitals installed hand-washing stations in emergency departments, and 5(26.3%) around the whole hospital. Out of 31 hospitals with no nosocomial SARS infection among HCWs, 28 (90.3%) hospitals installed hand-washing station in emergency departments (*P* < 0.001), and 20 (64.5%) hospitals had a set-up of hand-washing facilities around the whole hospital (*P* < 0.009) (31). The study concluded that the installation of a hand-washing station in emergency departments and around the whole hospital was significantly associated with effective prevention of nosocomial SARS infection during the SARS epidemic (31). Yu et al. (33) conducted a case-control hospital-based study (86 wards in 21 hospitals in Guangzhou and 38 wards in five hospitals in Hong Kong). Case wards were hospital wards in which super spreading events of SARS occurred (≥3 new cases of SARS) while control wards were hospital wards in which patients with SARS were admitted, but no subsequent outbreaks occurred. The study found that providing adequate washing or changing facilities for staff was protective [(OR, 0.12; 95% CI, 0.02–0.97), *P* = 0.05] for staff and helped reduce the risk of nosocomial outbreaks. This also submitted that HCWs could act as passive carriers of the SARS coronavirus, which would lead to nosocomial transmission. The study by Chen et al. (26) found that out of 748 frontline HCWs involved in the care of SARS patients in two hospitals in China; 91 HCWs developed SARS. The study also compared the frequency of washing hands, nasal cavity, and oral cavity after caring for SARS patients and found that frequently washes can prevent SARS transmission among HCWs.

##### Evidence on sterilization of hands

Studies also assessed the efficacy of sterilization of hands (29) and the availability of sterilization set-ups in hospitals (31) in controlling SARS. A case-control study conducted by Pei et al. in China, (29) found that out of 147 HCWs infected with SARS, 11 (7.5%) HCWs sterilized hands by iodine after contact with patients and out of 296 patients those did not develop SARS, 105 (39%) individuals sterilized hands by iodine after contact with patients [OR (95%CI): 0.14 (0.25–0.452), *P* = 0.00] (29). Another retrospective hospital-based study (33) from one of the epicenters of SARS in Taiwan found that out of 19 hospitals with one or more HCWs with a nosocomial SARS infection, 10 (52.6%) hospitals had a disinfectant solution available at the main entrance (of the hospital), and 5 (26.3%) hospitals had a set-up alcohol dispenser at checkpoints for glove-on hand rubbing between zones of risks. Out of 31 hospitals with no nosocomial SARS infection among HCWs, 30 (96.8%) hospitals had a disinfectant solution available at the main entrance (of the hospital) (*P* < 0.001), and had a set-up alcohol dispenser at checkpoints for glove-on hand rubbing between zones of risks (*P* < 0.001) (31). Stepwise logistic regression model of SARS prevention in hospitals of Taiwan found that set-up of alcohol dispensers at the checkpoint for glove-on hand rubbing between zones of risk was effective [OR (95%CI) 0.043 (0.003–0.627); *P* = 0.021] (31).

##### Evidence on nose wash

One study by Liu et al. (28) conducted a case-control study in China on 477 HCWs (representing 90% exposed to SARS patients) from Armed Forces Hospital with a nosocomial outbreak of SARS. The study found that out of 477 HCWs with SARS, 193 (40.46%) washed the nose. Reduction in ORs was achieved by washing the nose after attending to patients (*P* = 0.0002). The study thus concluded that nose washing was protective against infection. Also; significant correlations were found between performing nose wash and taking training (Correlation coefficient: 0.144, *P* = 0.004).

##### Evidence on gargling

One case-control study by Pei et al. (29) found that out of 147 HCWs infected with SARS, 9 (6.9%) HCWs did gargling and out of 296 patients did not develop SARS, 38 (13.5%) individuals did gargling after contact with patients [OR (95%CI): 0.474 (0.22–1.01), *P* = 0.049].

##### Evidence on cleaning/shower after duty

Two studies (29, 32) assessed the protective effects of cleaning after attending SARS patients. Case-control study by Pei et al. (29) found that out of 147 HCWs infected with SARS, 109 (82%) HCWs cleaned thoroughly after contact with patients and out of 296 patients that did not develop SARS, 261 (92.2%) cleaned thoroughly after contact with patients [OR (95%CI): 0.38 (0.20–0.71), *P* = 0.002] (29).

The study concluded that nosocomial infection of SARS can be avoided by adopting comprehensive protection measures (29). Another hospital-based case-control study by Yin et al. (32) in ten hospitals of China on HCWs involved in direct first aid for severe SARS patients found a dose-response relationship in taking shower and changing clothes after work (*P* < 0.01). The study also found that if more protective measures are used, the protective effect is higher (*P* < 0.001), and that the protective effect was 100% of all interventions were used at the same time.

##### Evidence on hospitalizations

None of the included studies reported data on hospitalizations of patients for symptoms suggestive of COVID-19 or SARS after WASH interventions as compared with no WASH interventions.

##### Evidence on mortality due to COVID-19 or SARS

None of the included studies reported data on mortality due to COVID-19 or SARS after WASH interventions as compared with no WASH interventions.

##### Evidence on adverse events due to WASH intervention

Only one retrospective hospital-based study (23) provided direct evidence of adverse events of excessive handwash caused by enhanced infection prevention measures in front-line HCWs during the COVID-19 pandemic. The study found that out of 542 front-line HCWs for COVID-19; 526 (97%) reported hand-skin damage due to enhanced infection-prevention measures. The study also demonstrated that longer exposure time was a significant risk factor as HCWs that washed their hands more than 10 times per day reported more hand-skin damage [OR (95%CI) = 2.17 (1.38–3.43), *P* = 0.01].

#### Comparison 2: WASH interventions vs. any other public health measures (without WASH interventions)

None of the included studies reported data on the effectiveness of WASH vs. any other public health measures (without WASH interventions) such as quarantine of individuals or a community, PPEs, physical distancing including lockdown, other workplace interventions; etc. on the number of COVID-19 or SARS cases, hospitalizations, mortality or any adverse events related to WASH.

##### GRADE assessment

We rated the certainty of evidence as very low for primary outcomes (number of cases). We downgraded one level due to high risk of bias in study design and twice for imprecision due to sparse data and low participant numbers (Table 4).

## Discussion

### Summary of main findings

To the best of our knowledge, this is the first rapid review of the effectiveness of WASH intervention to control COVID-19. The evidence base is limited because of the very direct few evidence on COVID-19. The other ten included studies are on SARS and contribute only indirect evidence. One study (25) reported the benefit of refined management strategies including hand hygiene and another study (24) reported the benefit of qualified and optimal hand hygiene practices in reducing the risk of COVID-19 among HCWs after coming in contact with infected patients. Other indirect evidence from previous SARS outbreak also suggests the benefit of hand hygiene, nose wash, gargling, shower and installation of handwash station, hand sanitizer station or shower facility in hospitals to avert transmission of SARS in HCWs. However, this evidence is based on the SARS outbreak, and generalizability to COVID-19 is very limited. In general, the combination of any of the WASH interventions with other prevention and control measures such as PPE, isolation, training, prophylactic medicines, proper ventilation, and other infection control measures had a greater effect on reducing the number of cases than individual measures.

### Overall completeness and applicability of the evidence

Person-to-person transmission of nCoV-2 has occurred in families, homes, colonies, hospitals, and between cities, states, countries, and continents. Many HCWs and other contacts of infected patients have been affected with COVID-19 after coming in contact with the infected patients. This has led to concern among workers and other contacts who are at risk of being infected while performing their duties. Looking at the current pattern of spread; public health measures and alternative medication are pressing management strategies against the COVID-19 pandemic (35). This pandemic has drawn the attention toward the importance of public health measures, such as personal hygiene, personal protective equipment, isolation of cases, quarantine, physical distancing, other workplace interventions; etc. Hygiene and Public Health are vital to a larger population (36).

SARS was the first pandemic of the twenty-first century which was ultimately brought under control through public health measures such as hygiene practices (e.g., frequent hand washing, face mask, and disinfecting living quarters), travel restraints, and quarantine (37). Infections among HCWs have been a common feature of SARS since it surfaced. It was observed that the majority of SARS cases occurred in settings where infection control measures had not been installed or established or had been installed or established but were not adhered to. CDC had recommended infection control measures such as careful hand hygiene, use of negative-pressure isolation rooms, N95 masks, gloves, gowns, and eye protection (38).

The generation of viral aerosol by a COVID-19 patient suggests a possibility of respiratory droplets transmission. The touchable surfaces in contact with infected patients can be contaminated by infected patients either through respiratory secretion or through hands. This underlines the necessity of suitable respiratory protection and also stringent surface hygiene practices. Conserving a hygienic environment can be one of the valuable public health measures to tackle such infectious diseases. Hand hygiene is a very simple and cost-effective public health infection control measure to prevent the spread of the infectious agent. Quarantine, hygiene measures, and protective equipment were the principal preventive measures that were found to be effective in limiting the spread of SARS in many countries (39). Suboptimal hand hygiene after contacting patients were linked to COVID-19 (24). The society also needs to be educated, supported, and prepared with the skills to foster better health and hygiene.

### Limitations in the body of evidence

We did not find any study that directly evaluated the effects of WASH alone or in combination with other measures to control COVID-19. Lack of data may be explained by the fact that the pandemic is still in progress, and such studies may be in progress. The majority of best available evidence in this review is from indirect evidence from nine case-control and one modeling study on SARS. Hence, the applicability and generalizability of evidence from studies on SARS is possibly limited because of different trajectories due to variations in transmission dynamics. Nevertheless, they back the findings for COVID-19.

### Potential biases in the review process

Due to the paucity of time, we conducted this rapid review and curtailed the steps adopted in systematic review methods and implemented some shortcuts in our methodology. We did not undertake searches of gray literature; or contacted experts for on-going studies or any authors for missing data. Moreover, we limited publications to the English language. As this pandemic is spreading rapidly, especially in countries like Italy and Spain, there remains a possibility that we may have missed studies conducted recently in these countries. During the screening of studies for eligibility criteria, the second reviewer checked 30% of the excluded records in the first phase and 100% of records in the second phase of screening. One reviewer conducted the 'Risk of bias' assessment, a second review author checked the acceptability and accuracy. This might have introduced some bias to this rapid review. However, in spite of these limitations, we are confident that none of these procedural curbs would have altered the general conclusions of this rapid review.

## Summary and perspective

### Implications for practice

Current evidence of WASH interventions for COVID-19 is limited due to lack of primary data on novel coronavirus infection and as is largely based on indirect evidence from SARS. Findings from the included studies consistently show that WASH is important in reducing the number of cases during the pandemic. Timely implementation of WASH along with other public health interventions can be vital to ensure better results. The policymakers will have to constantly supervise the pandemic situation and based upon the effect of the implemented public health measures in studies; suggest the best combination of public health interventions.

### Implications for research

Although the studies point toward the effectiveness of WASH interventions to control a pandemic, further good quality studies providing suitable, reliable and affordable direct evidence of the efficacy of WASH alone or in combination with other public health measures to control the cases and mortality due to COVID-19 as well as to control such public health emergencies are needed. Studies are also needed on the efficacy of sanitation (a component of WASH) alone or in combination with other public health measures to reduce the cases, hospitalizations, and mortality due to COVID-19 are also needed.

## Author contributions

MK contributed to the overall design, coordination, screening of studies against the eligibility criteria, assessment of RoB, and drafted the manuscript in consultation with the co-authors. AS contributed to the design, resolved discrepancy in screening of studies, and provided inputs on all drafts and the final version. GM overall manuscript drafting and reviewing. SQ contributed to the design, provided inputs related to statistics, and provided inputs on all drafts and the final version. SG contributed to the design and provided inputs on all drafts and the final version. DS and SS contributed to the design, screening of studies against the eligibility criteria, and provided inputs on all drafts and the final version. AG contributed to the design, co-ordination, assessed the certainty of the evidence, and provided inputs on all drafts and the final version. PB contributed to the design, verified the assessment of RoB, and provided inputs on all drafts and the final version. SS contributed reviewing the manuscript and provided inputs on all drafts and the final version. QZ contributed to the design, co-ordination, verified systematic literature searches, and drafted the manuscript in consultation with the MK, AS, GM, SQ, SG, DS, AG, PB, and SS. All authors contributed to the article and approved the submitted version.

## Funding

This article was self-funded by Datta Meghe Institute of Medical Sciences.

## Conflict of interest

The authors declare that the research was conducted in the absence of any commercial or financial relationships that could be construed as a potential conflict of interest.

## Publisher's note

All claims expressed in this article are solely those of the authors and do not necessarily represent those of their affiliated organizations, or those of the publisher, the editors and the reviewers. Any product that may be evaluated in this article, or claim that may be made by its manufacturer, is not guaranteed or endorsed by the publisher.
