# Supplementary material for: WASH to control COVID-19: A rapid review
Source: Front Public Health. 2022 Aug 11;10:976423. doi: 10.3389/fpubh.2022.976423 (PMC9403322; doi:10.3389/fpubh.2022.976423)
Supplement: Supplementary file 1 [file Table_1.pdf]

Supplementary Table 1: Search strategies

S.No	Databases	Search Strategies
1.	**MEDLINE via PubMed**	(((((((((covid*[Title/Abstract]) OR (coronavirus*[Title/Abstract])) OR ("corona virus*"[Title/Abstract])) OR (2019-nCoV[Title/Abstract])) OR (nCoV*[Title/Abstract])) OR (SARS*[Title/Abstract])) OR ("severe acute respiratory syndrome"[Title/Abstract])) OR (coronavirus, sars[MeSH Terms]))) AND ((((((WASH[Title/Abstract]) OR (wash*[Title/Abstract])) OR (hygiene[Title/Abstract])) OR (sanitation[Title/Abstract])) OR (sanitary[Title/Abstract]))))
2.	**CENTRAL**	ID Search<br>#1 coronavirus*<br>#2 "corona virus"<br>#3 nCoV*<br>#4 2019nCoV<br>#5 SARS-CoV-2<br>#6 MeSH descriptor: [Coronavirus] explode all trees<br>#7 COVID*<br>#8 #1 OR #2 OR #3 OR #4 OR #5 OR #6 OR #7<br>#9 WASH<br>#10 sanitation<br>#11 hygiene<br>#12 MeSH descriptor: [Sanitation] explode all trees<br>#13 MeSH descriptor: [Hygiene] explode all trees<br>#14 #9 OR #10 OR #11 OR #12 OR #13<br>#15 #8 AND #14
3.	**EMBASE**	#16 #9 AND #15<br>#15 OR #11 OR #12 OR #13 OR #14<br>#14 sanitary:ti,ab<br>#13 sanitation:ti,ab<br>#12 hygiene:ti,ab<br>#11 wash*:ti,ab<br>#10 wash:ti,ab<br>#9 #1 OR #2 OR #3 OR #4 OR #5 OR #6 OR #7 OR #8<br>#8 'sars coronavirus'/exp<br>#7 'severe acute respiratory syndrome':ti,ab<br>#6 sars*:ti,ab<br>#5 ncov*:ti,ab

		#4 '2019 ncov':ti,ab<br>#3 'corona virus*':ti,ab<br>#2 coronavirus*:ti,ab<br>#1 covid*:ti,ab
