# Supplementary material for: WASH to control COVID-19: A rapid review
Source: Front Public Health. 2022 Aug 11;10:976423. doi: 10.3389/fpubh.2022.976423 (PMC9403322; doi:10.3389/fpubh.2022.976423)
Supplement: Supplementary file 2 [file Table_2.pdf]

Supplemenatry File 2: List of excluded studies

SN	Excluded Study (ID)	Reason for exclusion
1.	Zhou and Jiang (1)	Article in Chinese
2.	Cochereau and Lamirel (2)	Article in French
3.	CDC (3)	Not on WASH
4.	Pinkas et al., (4)	Not an observational study
5.	File and Tsang (5)	Not an observational study
6.	Onishchenko (6)	Article in French
7.	Cooper et al., (7)	Not on WASH
8.	Wei et al., (8)	Not on WASH

References

1. Zhou ZX, Jiang CQ. [Effect of environment and occupational hygiene factors of hospital infection on SARS outbreak]. Zhonghua Lao Dong Wei Sheng Zhi Ye Bing Za Zhi. 2004 Aug;22(4):261–3.

2. Cochereau I, Lamirel C. Hygiène des mains et masques contre COVID-19 : oser le dégradé ? Journal Français d'Ophtalmologie. 2020 May;43(5):386–8.

3. Centers for Disease Control and Prevention (CDC). Cluster of severe acute respiratory syndrome cases among protected health-care workers--Toronto, Canada, April 2003. MMWR Morb Mortal Wkly Rep. 2003 May 16;52(19):433–6.

4. Pinkas J, Jankowski M, Szumowski Ł, Lusawa A, Zgliczyński WS, Raciborski F, et al. Public Health Interventions to Mitigate Early Spread of SARS-CoV-2 in Poland. Med Sci Monit [Internet]. 2020 Apr 13 [cited 2022 Jul 18];26. Available from: https://www.medscimonit.com/abstract/index/idArt/924730

5. File T, Tsang K. Severe Acute Respiratory Syndrome. 2005;4(2):12.

6. Onishchenko GG. [On the epidemiological situation in quarantine, natural focal and other infections on the territory of the Southern Federal District]. Zh Mikrobiol Epidemiol Immunobiol. 2004 Jun;(3):23–30.

7. Cooper BS, Fang LQ, Zhou JP, Feng D, Lv H, Wei MT, et al. Transmission of SARS in three Chinese hospitals. Tropical Medicine & International Health. 2009 Nov;14:71–8.

8. Wei MT, de Vlas SJ, Yang Z, Borsboom GJJM, Wang L, Li H, et al. The SARS outbreak in a general hospital in Tianjin, China: clinical aspects and risk factors for disease outcome. Tropical Medicine & International Health. 2009 Nov;14:60–70.
